# Entropy of Dynamical Social Networks

**DOI:** 10.1371/journal.pone.0028116

**Published:** 2011-12-16

**Authors:** Kun Zhao, Márton Karsai, Ginestra Bianconi

**Affiliations:** 1 Physics Department, Northeastern University, Boston, Massachusetts, United States of America; 2 Biomedical Engineering and Computational Science, School of Science, Aalto University, Aalto, Finland; University of Zaragoza, Spain

## Abstract

Human dynamical social networks encode information and are highly adaptive. To characterize the information encoded in the fast dynamics of social interactions, here we introduce the entropy of dynamical social networks. By analysing a large dataset of phone-call interactions we show evidence that the dynamical social network has an entropy that depends on the time of the day in a typical week-day. Moreover we show evidence for adaptability of human social behavior showing data on duration of phone-call interactions that significantly deviates from the statistics of duration of face-to-face interactions. This adaptability of behavior corresponds to a different information content of the dynamics of social human interactions. We quantify this information by the use of the entropy of dynamical networks on realistic models of social interactions.

## Introduction

Networks [1]–[5] encode information in the topology of their interactions. This is the main reason why networks are ubiquitous in complexity theory and constitute the underlying structures of social, technological and biological systems. The information encoded in social networks [6], [7] is essential to build strong collaborations [8] that enhance the performance of a society, to build reputation trust and to navigate [9] efficiently the networks. For these reasons social networks are small world [10] with short average distance between the nodes but large clustering coefficient. Therefore to understand how social network evolve, adapt and respond to external stimuli, we need to develop a new information theory of complex social networks.

Recently, attention has been addressed to entropy measures applied to email correspondence [11], static networks [12]–[14] and mobility patterns [15]. New network entropy measures quantify the information encoded in heterogenous static networks [12], [14]. Information theory tools set the limit of predictability of human mobility[15]. Still we lack methods to assess the information encoded in the dynamical social interaction networks.

Social networks are characterized by complex organizational structures revealed by network community and degree correlations [16]. These structures are sometimes correlated with annotated features of the nodes or of the links such as age, gender, and other annotated features of the links such as shared interests, family ties or common work locations [17], [18]. In a recent work [19] it has been shown by studying social, technological and biological networks that the network entropy measures can assess how significant are the annotated features for the network structure.

Moreover social networks evolve on many different time-scales and relevant information is encoded in their dynamics. In fact social networks are highly adaptive. Indeed social ties can appear or disappear depending on the dynamical process occurring on the networks such as epidemic spreading or opinion dynamics. Several models for adaptive social evolution have been proposed showing phase transitions in different universality classes [20]–[23]. Social ties have in addition to that a microscopic structure constituted by fast social interactions of the duration of a phone call or of a face-to-face interaction. Dynamical social networks characterize the social interaction at this fast time scale. For these dynamical networks new network measures are starting to be defined [24] and recent works focus on the implication that the network dynamics has on percolation, epidemic spreading and opinion dynamics [25]–[29].

Thanks to the availability of new extensive data on a wide variety of human dynamics [30]–[34], human mobility [15], [35], [36] and dynamical social networks [37], it has been recently recognized that many human activities [26] are bursty and not Poissonian. New data on social dynamical networks start to be collected with new technologies such as of Radio frequency Identification Devices [28], [38] and Bluetooth [31]. These technologies are able to record the duration of social interactions and report evidence for a bursty nature of social interaction characterized by a fat tail distribution of the duration of face-to face interactions. This bursty behavior of social networks [28], [38]–[42] is coexisting with modulations coming from periodic daily (circadian rhythms) or weakly patterns [43]. The fact that this bursty behavior is observed also in social interaction of simple animals, in the motion of rodents [44], or in the use of words [45], suggests that the underlying origin of this behavior is dictated by the biological and neurological processes underlying the dynamics of the social interaction. To our opinion this problem remains open: How much can humans intentionally change the statistics of social interactions and the level of information encoded in the dynamics of their social networks, when they are interfacing with a new technology?

In this paper we try to address this question by studying the dynamics of interactions through phone calls and comparing it with face-to-face interactions. We show that the entropy of dynamical networks is able to quantify the information encoded in the dynamics of phone-call interactions during a typical week-day. Moreover we show evidence that human social behavior is highly adaptive and that the duration of face-to-face interaction in a conference follows a different distribution than duration of phone-calls. We therefore have evidence of an intentional capability of humans to change statistically their behavior when interfacing with the technology of mobile phone communication. Finally we develop a model in order to quantify how much the entropy of dynamical networks changes if we allow modifications in the distribution of duration of the interactions.

## Results

### Entropy of dynamical social networks

**Figure 1:**
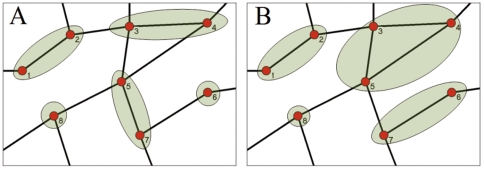
The dynamical social networks are composed by different dynamically changing groups of interacting agents. In panel (A) we allow only for groups of size one or two as it typically happens in mobile phone communication. In panel (B) we allow for groups of any size as in face-to-face interactions.

In this section we introduce the entropy of dynamical social networks as a measure of information encoded in their dynamics. Since we are interested in the dynamics of contacts we assume to have a quenched social network 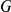 of friendships, collaborations or acquaintances formed by 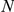 agents and we allow a dynamics of social interactions on this network. If two agents 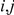 are linked in the network they can meet and interact at each given time giving rise to the dynamical social network under study in this paper. If a set of agents of size 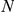 is connected through the social network 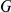 the agents 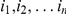 can interact in a group of size 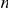. Therefore at any given time the static network 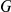 will be partitioned in connected components or groups of interacting agents as shown in Fig. 1. In order to indicate that a social interaction is occurring at time 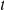 in the group of agents 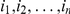 and that these agents are not interacting with other agents, we write 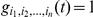 otherwise we put 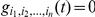. Therefore each agent is interacting with one group of size 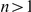 or non interacting (interacting with a group of size 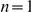). Therefore at any given time
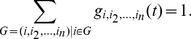
(1)where we indicate with 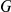 an arbitrary connected subgraph of 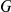. The history 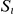 of the dynamical social network is given by 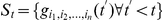. If we indicated by 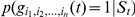 the probability that 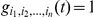 given the story 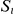, the likelihood that at time 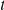 the dynamical networks has a group configuration 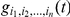 is given by
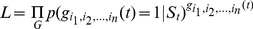
(2)


The entropy 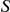 characterizes the logarithm of the typical number of different group configurations that can be expected in the dynamical network model at time 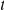 and is given by 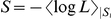 that we can explicitly express as
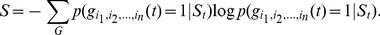
(3)According to the information theory results [46], if the entropy is vanishing, i.e. 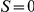 the network dynamics is regular and perfectly predictable, if the entropy is larger the number of future possible configurations is growing and the system is less predictable. If we model face-to-face interactions we have to allow the possible formation of groups of any size, on the contrary, if we model the mobile phone communication, we need to allow only for pairwise interactions. Therefore, if we define the adjacency matrix of the network 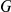 as the matrix 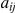, the log likelihood takes the very simple expression given by
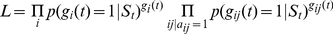
(4)with
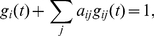
(5)for every time 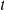. The entropy is then given by
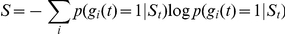


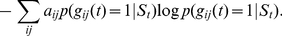
(6)


### Social dynamics and entropy of phone call interactions

We have analyzed the call sequence of subscribers of a major euroepan mobile service provider. We considered calls between users who at least once called each other during the examined 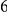 months period in order to examine calls only reflecting trusted social interactions. The resulted event list consists of 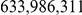 calls between 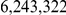 users. For the entropy calculation we selected 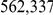 users who executed at least one call per a day during a week period. First of all we have studied how the entropy of this dynamical network is affected by circadian rhythms. We assign to each agent 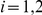 a number 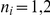 indicating the size of the group where he/she belongs. If an agent 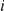 has coordination number 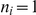 he/she is isolated, and if 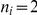 he/she is interacting with a group of 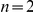 agents. We also assign to each agent 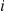 the variable 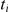 indicating the last time at which the coordination number 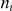 has changed. If we neglect the feature of the nodes, the most simple transition probabilities that includes for some memory effects present in the data, is given by a probability 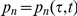 for an agent in state 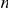 at time 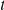 to change his/her state given that he has been in his/her current state for a duration 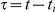.

**Figure 2:**
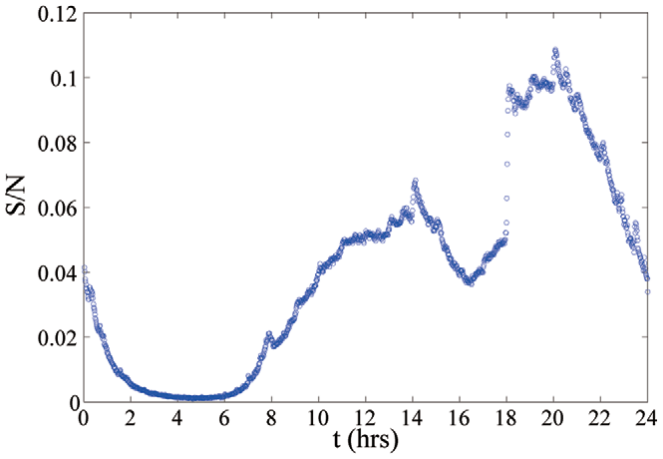
Mean-field evaluation of the entropy of the dynamical social networks of phone calls communication in a typical week-day. In the nights the social dynamical network is more predictable.

We have estimated the probability 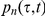 in a typical week-day. Using the data on the probabilities 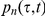 we have calculated the entropy, estimated by a mean-field evaluation (Check Text S1) of the dynamical network as a function of time in a typical week-day. The entropy of the dynamical social network is reported in Fig. 2. It significantly changes during the day describing the fact that the predictability of the phone-call networks change as a function of time. In fact, as if the entropy of the dynamical network is smaller and the network is an a more predictable state.

### Adaptive dynamics face-to face interactions and phone call durations

In this section we report evidence of adaptive human behavior by showing that the duration of phone calls, a binary social interactions mediated by technology, show different statistical features respect to face-to-face interactions. The distributions of the times describing human activities are typically broad [26], [28], [30], [32], [38], [39], and are closer to power-laws, which lack a characteristic time scale, than to exponentials. In particular in [38] there is reported data on Radio Frequency Identification devices, with temporal resolution of 20 s, showing that both distribution duration of face-to-face contacts and inter-contact periods is fat tailed during conference venues.

**Figure 3:**
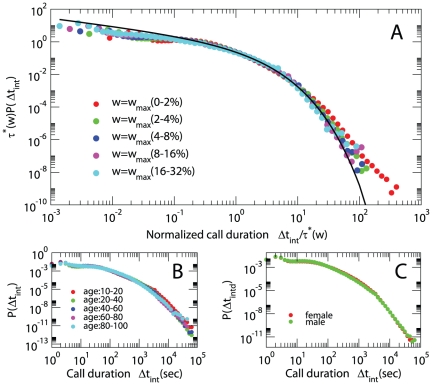
Probability distribution of duration of phone-calls. (A) Probability distribution of duration of phone-calls between two given persons connected by a link of weight 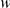. The data depend on the typical scale 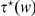 of duration of the phone-call. (B) Probability distribution of duration of phone calls for people of different age. (C) Probability distribution of duration of phone-calls for people of different gender. The distributions shown in the panel (B) and (C) do not significantly depend on the attributes of the nodes.

**Figure 4:**
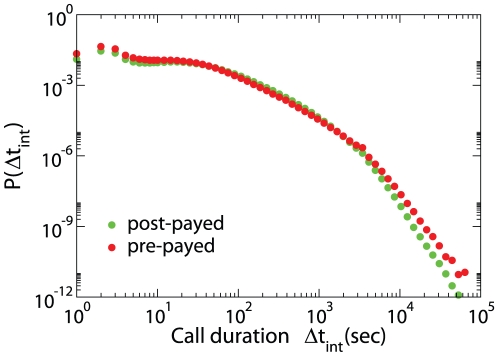
Probability distribution of duration of phone-calls for people with different types of contract. No significant change is observed that modifies the functional form of the distribution.

**Figure 5:**
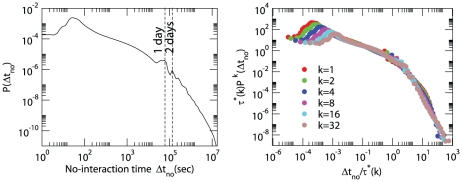
Distribution of non-interaction times in the phone-call data. The distribution strongly depends on circadian rhythms. The distribution of rescaled time depends strongly on the connectivity of each node. Nodes with higher connectivity 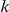 are typically non-interacting for a shorter typical time scale 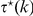.

Here we analysed the above defined mobile-call event sequence performing the measurements on all the users for the entire 6 months time period. The distribution of phone-call durations strongly deviates from a fat-tail distribution. In Fig. 3 we report this distributions and show that these distributions depend on the strength 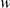 of the interactions (total duration of contacts in the observed period) but do not depend on the age, gender or type of contract in a significant way. The distribution 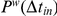 of duration of contacts within agents with strenght 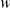 is well fitted by a Weibull distribution
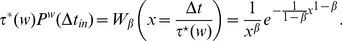
(7)with 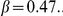. The typical times 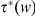 used for the data collapse of Figure 3 are listed in Table 1. The origin of this significant change in behavior of humans interactions could be due to the consideration of the cost of the interactions (although we are not in the position to draw these conclusions (See Fig. 4 in which we compare distribution of duration of calls for people with different type of contract) or might depend on the different nature of the communication. The duration of a phone call is quite short and is not affected significantly by the circadian rhythms of the population. On the contrary the duration of no-interaction periods is strongly affected by periodic daily of weekly rhythms. The distribution of no-interaction periods can be fitted by a double power-law but also a single Weibull distribution can give a first approximation to describe 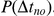 In Fig. 5 we report the distribution of duration of no-interaction periods in the day periods between 7AM and 2AM next day. The typical times 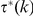 used in Figure 5 are listed in Table 2.

**Table 1:** Typical times 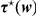 used in the data collapse of Fig. 3.

Weight of the link	Typical time 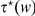 in seconds (s)
(0–2%) 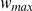	111.6
(2–4%) 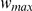	237.8
(4–8%) 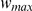	334.4
(8–16%) 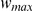	492.0
(16–32%) 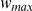	718.8

**Table 2:** Typical times 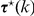 used in the data collapse of Fig. 5.

Connectivity	Typical time 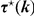 in seconds (s)
k = 1	158,594
k = 2	118,047
k = 4	69,741
k = 8	39,082
k = 16	22,824
k = 32	13,451

## Discussion

### The entropy of a realistic model of cell-phone interactions

The data on face-to-face and mobile-phone interactions show that a reinforcement dynamics is taking place during the human social interaction. Disregarding for the moment the effects of circadian rhythms and weakly patterns, a possible explanation of such results is given by mechanisms in which the decisions of the agents to form or leave a group are driven by memory effects dictated by reinforcement dynamics, that can be summarized in the following statements: *i) the longer an agent is interacting in a group the smaller is the probability that he/she will leave the group; ii) the longer an agent is isolated the smaller is the probability that he/she will form a new group*. In particular, such reinforcement principle implies that the probabilities 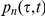 that an agent with coordination number 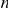 changes his/her state depends on the time elapsed since his/her last change of state, i.e., 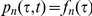. To ensure the reinforcement dynamics any function 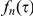 which is a decreasing function of its argument can be taken. In two recent papers[41], [42] the face-to-face interactions have been realistically modelled with the use of the reinforcement dynamics, by choosing
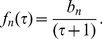
(8)with good agreement with the data when we took 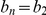 for 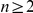 and 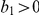, 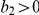.

In order to model the phone-call data studied in this paper we can always adopt the reinforcement dynamics but we need to modify the probability 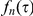 by a parametrization with an additional parameter 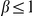. In order to be specific in our model of mobile-phone communication, we consider a system that consists of 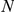 agents. Corresponding to the mechanism of daily cellphone communication, the agents can call each other to form a binary interaction if they are neighbor in the social network. The social network is characterized by a given degree distribution 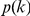 and a given weight distribution 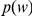. Each agent 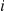 is characterized by the size 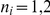 of the group he/she belongs to and the last time 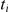 he/she has changed his/her state. Starting from random initial conditions, at each timestep 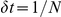 we take a random agent. If the agent is isolated he/she will change his/her state with probability
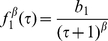
(9)with 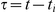 and 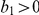. If he/she change his/her state he/she will call one of his/her neighbor in the social network which is still not engaged in a telephone call. A non-interacting neighbor agent will pick up the phone with probability 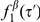 where 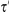 is the time he/she has not been interacting.

If, on the contrary the agent 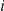 is interacting, he/she will change his/her state with probability 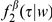 depending on the weight of the link and on the duration of the phone call. We will take in particular
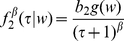
(10)where 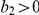 and 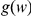 is a decreasing function of the weight 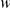 of the link. The distributions 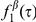 and 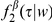 are parametrized by the parameter 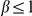. As 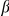 increases, the distribution of duration of contacts and duration of intercontact time become broader. These probabilities give rise to either Weibull distribution of duration of interactions (if 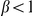) or power-law distribution of duration of interaction 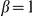. Indeed for 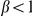, the probability 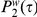 that a conversation between two nodes with link weight 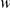 ends after a duration 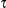 is given by the Weibull distribution (See Text S1 for the details of the derivation)
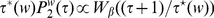
(11)with 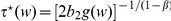. This distribution well capture the distribution observed in mobile phone data and reported in Fig. 3 (for a discussion of the validity of the annealed approximation for predictions on a quenched network see the Text S1 ).

If, instead of having 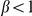 we have 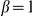 the probability distribution for duration of contacts is given by a power-law
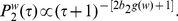
(12)This distribution is comparable with the distribution observed in face-to-face interaction during conference venues [41], [42]. The adaptability of human behavior, evident when comparing the distribution of duration of phone-calls with the duration of face-to-face interactions, can be understood as a possibility to change the exponent 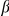 regulating the duration of social interactions.

**Figure 6:**
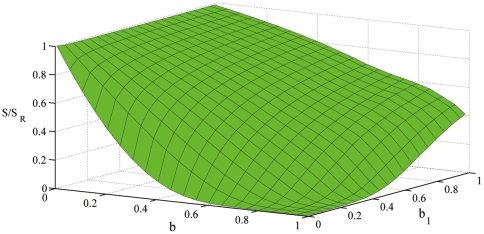
Entropy 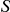 of social dynamical network model of pairwise communication normalized with the entropy 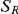 of a null model in which the expected average duration of phone-calls is the same but the distribution of duration of phone-calls and non-interaction time are Poisson distributed. The network size is 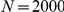 the degree distribution of the network is exponential with average 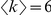, the weight distribution is 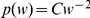 and 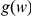 is taken to be 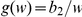 with 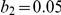. The value of 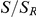 is depending on the two parameters 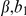. For every value of 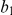 the normalized entropy is smaller for 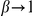.

Changes in the parameter 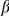 correspond to a different entropy of the dynamical social network. Solving analytically this model we are able to evaluate the dynamical entropy as a function of 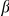 and 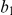. In Fig. 6 we report the entropy 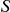 of the dynamical social network a function of 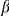 and 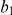 in the annealed approximation and the large network limit. In particular we have taken a network of size 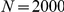 with exponential degree distribution of average degree 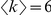, weight distribution 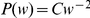 and function 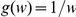 and 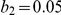. Our aim in Fig. 6 is to show only the effects on the entropy due to the different distributions of duration of contacts and non-interaction periods. Therefore we have normalized the entropy 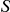 with the entropy 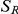 of a null model of social interactions in which the duration of groups are Poisson distributed but the average time of interaction and non interaction time are the same as in the model of cell-phone communication. From Fig. 6 we observe that if we keep 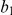 constant, the ratio 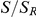 is a decreasing function of the parameter 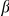 indicating that the broader are the distribution of probability of duration of contacts the higher is the information encoded in the dynamics of the networks. Therefore the heterogeneity in the distribution of duration of contacts and no-interaction periods implies higher level of information in the social network. The human adaptive behavior by changing the exponent 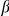 in face-to-face interactions and mobile phone communication effectively change the entropy of the dynamical network.

In conclusion, in the last ten years it has been recognized that the vast majority of complex systems can be described by networks of interacting units. Network theory has made tremendous progresses in this period and we have gained important insight into the microscopic properties of complex networks. Key statistical properties have been found to occur universally in the networks, such as the small world properties and broad degree distributions. Moreover the local structure of networks has been characterized by degree correlations, clustering coefficient, loop structure, cliques, motifs and communities. The level of information present in these characteristic of the network can be now studied with the tools of information theory. An additional fundamental aspect of social networks is their dynamics. This dynamics encode for information and can be modulated by adaptive human behavior. In this paper we have introduced the entropy of social dynamical networks and we have evaluated the information present in dynamical data of phone-call communication. By analysing the phone-call interaction networks we have shown that the entropy of the network depends on the circadian rhythms. Moreover we have shown that social networks are extremely adaptive and are modified by the use of technologies. The statistics of duration of phone-call indeed is described by a Weibull distribution that strongly differ from the distribution of face-to-face interactions in a conference. Finally we have evaluated how the information encoded in social dynamical networks change if we allow a parametrization of the duration of contacts mimicking the adaptability of human behavior. Therefore the entropy of social dynamical networks is able to quantify how the social networks dynamically change during the day and how they dynamically adapt to different technologies.

## Materials and Methods

In order to describe the model of mobile phone communication, we consider a system consisting of 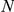 agents representing the mobile phone users. The agents are interacting in a social network 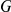 representing social ties such as friendships, collaborations or acquaintances. The network 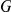 is weighted with the weights indicating the strength of the social ties between agents. We use 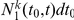 to denote the number of agents with degree 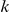 that at time 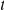 are not interacting and have not interacted with another agent since time 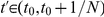. Similarly we denote by 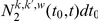 the number of connected agents (with degree respectively 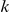 and 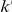 and weight of the link 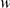) that at time 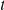 are interacting in phone call started at time 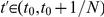. The mean-field equation for this model read,
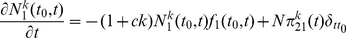


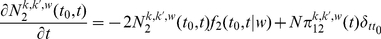
(13)where the constant 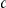 is given by
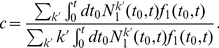
(14)In Eqs. 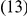 the rates 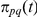 indicate the average number of agents changing from state 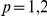 to state 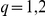 at time 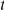. These rates can be also expressed in a self-consistent way and the full system solved for any given choice of 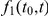 and 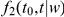 (See Text S1 for details).

The definition of the entropy of dynamical social networks of a pairwise communication model, is given by Eq. (6). To evaluate the entropy of dynamical social network explicitly, we have to carry out the summations in Eq. 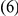. These sums, will in general depend on the particular history of the dynamical social network, but in the framework of the model we study, in the large network limit will be dominated by their average value. In the following therefore we perform these sum in the large network limit. The first summation in Eq. 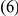 denotes the average loglikelihood of finding at time 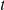 a non-interacting agent given a history 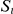. We can distinguish between two eventual situations occurring at time 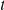: *(i)* the agent has been non-interacting since a time 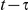, and at time 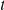 remains non-interacting; *(ii)* the agent has been interacting with another agent since time 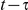, and at time 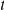 the conversation is terminated by one of the two interacting agents.The second term in the right hand side of Eq. 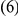, denotes the average loglikelihood of finding two agents in a connected pair at time 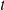 given a history 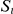. There are two possible situations that might occur for two interacting agents at time 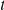: *(iii)* these two agents have been non-interacting, and to time 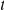 one of them decides to form a connection with the other one; *(iv)* the two agents have been interacting with each other since a time 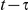, and they remain interacting at time 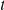. Taking into account all these possibilities we have been able to use the transition probability form different state and the number of agents in each state to evaluate the entropy of dynamical networks in the large network limit (For further details on the calculation see the Text S1).

### Ethics Statement

The dataset used in this study only involved de-identified information and no details about the subscribers were made available to us.

## Supporting Information

Text S1 **Supporting Information.**
(PDF)
