# Supplementary material for: Entropy of Dynamical Social Networks
Source: PLoS One. 2011 Dec 16;6(12):e28116. doi: 10.1371/journal.pone.0028116 (PMC3241622; doi:10.1371/journal.pone.0028116)
Supplement: Text S1 — Supporting Information. (PDF) [file pone.0028116.s001.pdf]

Text S1- Supporting Information of the paper ”Entropy of dynamical networks” by K. Zhao, M. Karsai and G. Bianconi

1 The proposed model of cellphone communication

1.1 Dynamical social network for pairwise communication

We consider a system consisting of N agents representing the mobile phone users. The agents are interacting in a social network G representing social ties such as friendships, collaborations or acquaintances. The network G is weighted with the weights indicating the strength of the social ties between agents. To model the mechanism of cellphone communication, the agents can call their neighbors in the social network G forming groups of interacting agents of size two. Since at any given time a call can be initiated or terminated the network is highly dynamical. We assign to each agent $i = 1, 2, \ldots, N$ a coordination number n_i to indicate his/her state. If $n_i = 1$ the agent is non-interacting, and if $n_i = 2$ the agent is in a mobile phone connection with another agent. The dynamical process of the model at each time step t can be described explicitly by the following algorithm:

(1) An agent i is selected randomly at time t.

(2) The subsequent action of agent i depends on his/her current state (i.e. n_i):

   (i) If $n_i = 1$, he/she will call one of his/her non-interacting neighbors j of G with probability $f_1(t_i, t)$ where t_i denotes the last time at which agent i has changed his/her state. Once he/she decides to call, agent j will be chosen randomly in between the neighbors of i with probability proportional to $f_1(t_j, t)$, therefore the coordination numbers of agent i and j are updated according to the rule $n_i \to 2$ and $n_j \to 2$.

   (ii) If $n_i = 2$, he/she will terminate his/her current connection with probability $f_2(t_i, t|w_{ij})$ where w_{ij} is the weight of the link between i and the neighbor j that is interacting with i. Once he/she decides to terminate the connection, the coordination numbers are then updated according to the rule $n_i \to 1$ and $n_j \to 1$.

(3) Time t is updated as $t \to t + 1/N$ (initially $t = 0$) and the process is iterated until $t = T_{max}$.

1.2 General solution to the model

In order to solve the model analytically, we assume the quenched network G to be annealed and uncorrelated. Therefore we assume that at each time the network is rewired keeping the degree distribution $p(k)$ and the weight distribution $p(w)$ constant. Moreover we solve the model in the continuous time limit.Therefore we always approximate the sum over time-steps of size $\delta t = 1/N$ by integrals over time. We use $N_1^k(t_0,t)dt_0$ to denote the number of agents with degree k that at time t are not interacting and have not interacted with another agent since time $t' \in (t_0, t_0+1/N)$. Similarly we denote by $N_2^{k,k',w}(t_0,t)dt_0$ the number of connected agents (with degree respectively k and k' and weight of the link w) that at time t are interacting in phone call started at time $t' \in (t_0, t_0+1/N)$. Consistently with the annealed approximation the probability that an agent with degree k is called is proportional to its degree. Therefore the rate equations of the model are given by

$$\begin{aligned}
\frac{\partial N_1^k(t_0,t)}{\partial t} &= -N_1^k(t_0,t)f_1(t_0,t) - ckN_1^k(t_0,t)f_1(t_0,t) + N\pi_{21}^k(t)\delta_{tt_0} \\
\frac{\partial N_2^{k,k',w}(t_0,t)}{\partial t} &= -2N_2^{k,k',w}(t_0,t)f_2(t_0,t|w) + N\pi_{12}^{k,k',w}(t)\delta_{tt_0}
\end{aligned} \tag{1}$$

where the constant c is given by

$$c = \frac{\sum_{k'} \int_0^t dt_0 N_1^{k'}(t_0,t)f_1(t_0,t)}{\sum_{k'} k' \int_0^t dt_0 N_1^{k'}(t_0,t)f_1(t_0,t)}. \tag{2}$$

In Eqs. (1) the rates $\pi_{pq}(t)$ indicate the average number of agents changing from state $p = 1,2$ to state $q = 1,2$ at time t. These rates can be also expressed in a self-consistent way as

$$\begin{aligned}
\pi_{21}^k(t) &= \frac{2}{N}\sum_{k',w}\int_0^t dt_0 f_2(t_0,t|w)N_2^{k,k',w}(t_0,t) \\
\pi_{12}^{k,k',w}(t) &= \frac{P(w)}{CN}\int_0^t dt_0 \int_0^t dt_0' N_1^k(t_0,t)N_1^{k'}(t_0',t)f_1(t_0,t)f_1(t_0',t)(k+k')
\end{aligned} \tag{3}$$

where the constant C is given by

$$C = \sum_{k'} \int_0^t dt_0 k' N_1^{k'}(t_0,t)f_1(t_0,t). \tag{4}$$

The solution to Eqs. (1) is given by

$$\begin{aligned}
N_1^k(t_0,t) &= N\pi_{21}^k(t_0)e^{-(1+ck)\int_{t_0}^t f_1(t_0,t)dt} \\
N_2^{k,k',w}(t_0,t) &= N\pi_{12}^{k,k',w}(t_0)e^{-2\int_{t_0}^t f_2(t_0,t|w)dt}
\end{aligned} \tag{5}$$

which must satisfy the self-consistent constraints Eqs. (3) and the conservation of the number of agents with different degree

$$\int dt_0 \big[N_1^k(t_0,t) + \sum_{k',w} N_2^{k,k',w}(t_0,t)\big] = Np(k). \tag{6}$$

In the following we will denote by $P_1^k(t_0,t)$ the probability distribution that an agent with degree k is non-interacting for a period from t_0 to t and by $P_2^w(t_0,t)$ the probability that a connection of weight w at time t is active since time t_0. It is immediate to see that these distributions are given by the number of individual in a state $n=1,2$ multiplied by the probability of having a change of state, i.e.

$$\begin{aligned} P_1^k(t_0,t) &= (1+ck)f_1(t_0,t)N_1^k(t_0,t) \\ P_2^w(t_0,t) &= 2f_2(t_0,t|w)\sum_{k,k'} N_2^{k,k',w}(t_0,t). \end{aligned} \tag{7}$$

1.3 Stationary solution with specific $f_1(t_0,t)$ and $f_2(t_0,t)$

In order to capture the behavior of the empirical data with a realistic model, we have chosen

$$\begin{aligned} f_1(t_0,t) &= f_1(\tau) = \frac{b_1}{(1+\tau)^\beta} \\ f_2(t_0,t|w) &= f_2(\tau|w) = \frac{b_2 g(w)}{(1+\tau)^\beta} \end{aligned} \tag{8}$$

with parameters $b_1 > 0$, $b_2 > 0$, $0 \leq \beta \leq 1$ and arbitrary positive function $g(w)$. In Eqs. (8), τ is the duration time elapsed since the agent has changed his/her state for the last time (i.e. $\tau = t - t_0$). The functions of $f_1(\tau)$ and $f_2(\tau|w)$ are decreasing function of their argument τ reflecting the reinforcement dynamics discussed in the main body of the paper. The function $g(w)$ is generally chosen as a decreasing function of w, indicating that connected agents with a stronger weight of link interact typically for a longer time. We are especially interested in the stationary state solution of the dynamics. In this regime we have that for large times $t \gg 1$ the distribution of the number of agents is only dependent on τ. Moreover the transition rates $\pi_{pq}(t)$ also converge to a constant independent of t in the stationary state. Therefore the solution of the stationary state will satisfy

$$\begin{aligned} N_1^k(t_0,t) &= N_1^k(\tau) \\ N_2^{k,k',w}(t_0,t) &= N_2^{k,k',w}(\tau) \\ \pi_{pq}(t) &= \pi_{pq}. \end{aligned} \tag{9}$$

The necessary condition for the stationary solution to exist is that the summation of self-consistent constraints given by Eq. (2) and Eq. (4) together with the conservation law Eq. (6) converge under the stationary assumptions Eqs. (9). The convergence depends

on the value of the parameters b_0, b_1, β and the choice of function $g(w)$. In particular, when $0 \leq \beta < 1$, the convergence is always satisfied. In the following subsections, we will characterize further the stationary state solution of this model in different limiting cases.

1.3.1 Case $0 < \beta < 1$

The expression for the number of agent in a given state $N_1^k(\tau)$ and $N_2^{k,k',w}(\tau)$ can be obtained by substituting Eqs. (8) into the general solution Eqs. (5), using the stationary conditions Eqs. (9). In this way we get the stationary solution given by

$$N_1^k(\tau) = N\pi_{21}^k e^{\frac{b_1(1+ck)}{1-\beta}[1-(1+\tau)^{1-\beta}]} = N\pi_{21}^k m_1^k(\tau)$$
$$N_2^{k,k',w}(\tau) = N\pi_{12}^{k,k',w} e^{\frac{2b_2 g(w)}{1-\beta}[1-(1+\tau)^{1-\beta}]} = N\pi_{12}^{k,k',w} m_2^w(\tau). \tag{10}$$

To complete the solution is necessary to determine the constants π_{21}^k and $\pi_{12}^{k,k'w}$ in a self-consistent type of solution.To find the expression of $\pi_{12}^{k,k',w}$ as a function of π_{21}^k we substitute Eqs. (10) in Eq.(3) and we get

$$\begin{aligned}\pi_{12}^{k,k',w}(t) &= \frac{1}{C}\pi_{21}^k P(w)\Big[k\int_0^t dt_0 m_1^k(t_0,t) f_1(t_0,t)\int_0^t dt'_0 N_1^{k'}(t'_0,t) f_1(t'_0,t)\\ &+ k'\int_0^t dt_0 m_1^k(t_0,t) f_1(t_0,t)\int_0^t dt'_0 N_1^{k'}(t'_0,t) f_1(t'_0,t)\Big].\end{aligned} \tag{11}$$

Finally we get a closed equation for π_{21}^k by substituting Eq.(11) in Eq.(6) and using the definition of c and C, given respectively by Eq. (2) and Eq. (4). Therefore we get

$$\begin{aligned}\pi_{21}^k \quad & \Big[\int_0^\infty m_1^k(\tau)d\tau + \int_{w_{min}}^{w_{max}} P(w)\int_0^\infty m_2^w(\tau)d\tau dw\\ \times & \left(ck\int_0^\infty m_1^k(\tau)f_1(\tau)d\tau + \int_0^\infty m_1^k(\tau)f_1(\tau)d\tau\right)\Big] = p(k).\end{aligned} \tag{12}$$

Performing explicitly the last two integrals using the dynamical solution given by Eqs. (10), this equation can be simplified as

$$\pi_{21}^k = \left[\int_0^\infty m_1^k(\tau)d\tau + \int_{w_{min}}^{w_{max}} P(w)\int_0^\infty m_2^w(\tau)d\tau dw\right]^{-1} p(k). \tag{13}$$

Finally the self-consistent solution of the dynamics is solved by expressing Eq. (2) by

$$c = \frac{\sum_k \pi_{21}^k(1+ck)^{-1}}{\sum_k \pi_{21}^k k(1+ck)^{-1}}. \tag{14}$$

Therefore we can use Eqs. (13) and (14) to compute the numerical value of π_{21}^k and c. Inserting in these equations the expressions for $f_1(\tau), f_2(\tau|w)$ given by Eqs. (8) and the

solutions $N_1^k(\tau), N_2^{k,k',w}(\tau)$ given by Eqs. (10) we get

$$\begin{aligned} P_1^k(\tau) &\propto \frac{b_1(1+ck)}{(1+\tau)^\beta} e^{-\frac{b_1(1+ck)}{1-\beta}(1+\tau)^{1-\beta}} \\ P_2^w(\tau) &\propto \frac{2b_2 g(w)}{(1+\tau)^\beta} e^{-\frac{2b_2 g(w)}{1-\beta}(1+\tau)^{1-\beta}}. \end{aligned} \quad (15)$$

The probability distributions $P_1^k(\tau)$ and $P_2^w(\tau)$, can be manipulating performing a data collapse of the distributions, i.e.

$$\begin{aligned} \tau_1^\star(k) P_1^k\left(x_1 = \frac{\tau}{\tau_1^\star(k)}\right) &= A_1 {x_1}^{-\beta} e^{-\frac{{x_1}^{1-\beta}}{1-\beta}} \\ \tau_2^\star(w) P_2^w\left(x_2 = \frac{\tau}{\tau_2^\star(w)}\right) &= A_2 {x_2}^{-\beta} e^{-\frac{{x_2}^{1-\beta}}{1-\beta}} \end{aligned} \quad (16)$$

with $\tau_1^\star(k)$ and $\tau_2^\star(w)$ defined as

$$\begin{aligned} \tau_1^\star(k) &= \left[b_1(1+ck)\right]^{-\frac{1}{1-\beta}} \\ \tau_2^\star(w) &= \left[2b_2 g(w)\right]^{-\frac{1}{1-\beta}} \end{aligned} \quad (17)$$

where A_1 and A_2 are the normalization factors. The data collapse defined by Eqs. (16) of the curves $P_1^k(\tau)$, $P_2^w(\tau)$ and are both described by Weibull distributions.

1.4 Comparisons with quenched simulations

To check the validity of our annealed approximation versus quenched simulations, we performed a computer simulation according to the dynamical process on a quenched network. In Fig. 1 we compare the results of the simulation with the prediction of the analytical solution. In particular in the reported simulation we have chosen $\beta = 0.5$, $b_1 = 0.02$, $b_2 = 0.05$ and $g(w) = w^{-1}$, the simulation is based on a number of agent $N = 2000$ and for a period of $T_{max} = 10^5$, finally the data are averaged over 10 realizations and the network is Poisson with average $\langle k \rangle = 6$ and weight distribution $p(w) \propto w^{-2}$. In Fig. 1, we show evidence that the Weibull distribution and the data collapse of $P_2^w(\tau)$ well capture the empirical behavior observed in the mobile phone data (Fig. **??**). The distribution of the non-interaction periods $P_1^k(\tau)$ in the model is by construction unaffected by circadian rhythms but follow a similar data collapse as observed in the real data (Fig. **??**). The simulated data are also in good agreement with the analytical prediction predicted in the annealed approximation for the parameter choosen in the figure. As the network becomes more busy and many agents are in a telephone call, the quenched simulation and the annealed prediction of $P_1^k(\tau)$ differs more significantly.

1.4.1 Case $\beta = 0$

For $\beta = 0$ the functions $f_1(\tau)$ and $f_2(\tau|w)$ given by Eqs.(8) reduce to constants, therefore the process of creation of an interaction is a Poisson process and no reinforcement

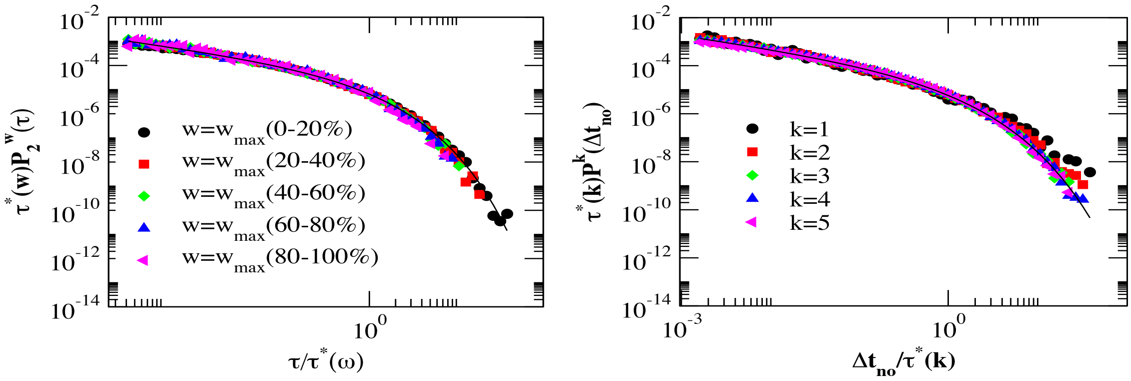

Figure 1: Data collapse of the simulation of the proposed model for cell phone communication. In the panel (A) we plot the probability $P_2^w(\tau)$ that in the model a pair of agents with strenght w are interacting for a period τ and in the panel (B) we plot the probability $P_1^k(\tau)$ that in the model an agents of degree k is non-interacting for a period τ The simulation data on a quenched networks are compared with the analytical predictions (solid lines) in the annealed approximation. The collapses data of $P_2^w(\tau)$ is described by Weibull distribution in agreement with the empirical results found in the mobile phone data.

dynamics is taking place in the network. Assigning $\beta = 0$ to Eqs. (5), we get the solution

$$
\begin{aligned}
N_1^k(\tau) &= N\pi_{21}^k e^{-b_1(1+ck)\tau} \\
N_2^{k,k',w}(\tau) &= N\pi_{12}^{k,k',w} e^{-2b_2 g(w)\tau}.
\end{aligned} \tag{18}
$$

and consequently the distributions of duration of given states Eqs. (7) are given by

$$
\begin{aligned}
P_1^k(\tau) &\propto e^{-b_1(1+ck)\tau} \\
P_2^w(\tau) &\propto e^{-2b_2 g(w)\tau}.
\end{aligned} \tag{19}
$$

Therefore the probability distributions $P_1^k(\tau)$ and $P_2^w(\tau)$ are exponentials as expected in a Poisson process.

1.4.2 Case $\beta = 1$

In this section, we discuss the case for $\beta = 1$ such that $f_1^k(\tau) \propto (1+\tau)^{-1}$ and $f_2^w(\tau|w) \propto (1+\tau)^{-1}$. Using Eqs. (1) we get the solution

$$
\begin{aligned}
N_1^k(\tau) &= N\pi_{21}^k (1+\tau)^{-b_1(1+ck)} \\
N_2^{k,k',w}(\tau) &= N\pi_{12}^{k,k',w} (1+\tau)^{-2b_2 g(w)}.
\end{aligned} \tag{20}
$$

and consequently the distributions of duration of given states Eqs. (7) are given by

$$
\begin{aligned}
P_1^k(\tau) &\propto \pi_{21}^k (1+\tau)^{-b_1(1+ck)-1} \\
P_2^w(\tau) &\propto \pi_{12}^{k,k',w} (1+\tau)^{-2b_2 g(w)-1}.
\end{aligned} \tag{21}
$$

The probability distributions are power-laws.This result remains valid for every value of the parameters $b_1, b_2, g(w)$ (See Ref. [1] for a full account of the detailed solution of this model) nevertheless the stationary condition is only valid for

$$\begin{aligned} b_1(1+ck) &> 1 \\ 2b_2 g(w) &> 1. \end{aligned} \tag{22}$$

Indeed this condition ensures that the self-consistent constraits Eqs. (2), (4) and the conservation law Eq. (6) have a stationary solution.

1.5 Solution of the mean-field model on a fully connected network

Finally, we discuss the mean-field limit on the model in which every agent can interact with every other agent. In this case, social network is a fully connected network. Therefore we use $N_1(t_0, t)$ and $N_2(t_0, t)$ to denote the number of agents of the two different states respectively and the rate equations are then revised to

$$\begin{aligned} \frac{\partial N_1(t_0,t)}{\partial t} &= -2N_1(t_0,t)f_1(t_0,t) + N\pi_{21}(t)\delta_{tt_0} \\ \frac{\partial N_2(t_0,t)}{\partial t} &= -2N_2(t_0,t)f_2(t_0,t) + N\pi_{12}(t)\delta_{tt_0} \end{aligned} \tag{23}$$

Since we will refer to this model only in the framework of a null model, we will only discuss the case in which the dynamics of the network is Poissonian, i.e. when

$$\begin{aligned} f_1(t_0,t) &= b_1 \\ f_2(t_0,t) &= b_2. \end{aligned} \tag{24}$$

The stationary solution of this model is given by exponentials, i.e.

$$\begin{aligned} N_1(\tau) &= N\pi_{21}e^{-2b_1\tau} \\ N_2(\tau) &= N\pi_{12}e^{-2b_2\tau}. \end{aligned} \tag{25}$$

Finally the distributions of duration of given states expressed by Eqs. (7) are given by

$$\begin{aligned} P_1(\tau) &\propto e^{-2b_1\tau} \\ P_2(\tau) &\propto e^{-2b_2\tau}, \end{aligned} \tag{26}$$

which are exponential distributions as expected in a Poisson process.

2 Entropy of the dynamical social networks

2.1 Entropy of the dynamical social networks of pairwise communication

The definition of the entropy of dynamical social networks of a pairwise communication model, is given by Eq. (6) of the main body of the article that we repeat here for convenience,

$$
\begin{aligned}
S = & -\sum_i P(g_i(t)=1|\mathcal{S}_t)\log P(g_i(t)=1|\mathcal{S}_t) \\
& -\sum_{ij} a_{ij} P(g_{ij}(t)=1|\mathcal{S}_t)\log P(g_{ij}(t)=1|\mathcal{S}_t)
\end{aligned}
\quad (27)
$$

In this equation the matrix a_{ij} is the adjacency matrix of the social network and $g_{ij}(t)=1$ indicates that at time t the agents i and j are interacting while $g_i(t)=1$ indicates that agent i is non-interacting.Finally $\mathcal{S}_t=\{g_i(t'), g_{ij}(t') \quad \forall t'<t\}$ indicates the dynamical evolution of the social network. In this section, we will evaluate the entropy of dynamical social networks in the framework of the annealed model of pairwise communication explained in detail in the previous section of this supplementary material. To evaluate the entropy of dynamical social network explicitly, we have to carry out the summations in Eq. (27). These sums, will in general depend on the particular history of the dynamical social network, but in the framework of the model we study, in the large network limit will be dominated by their average value. In the following therefore we perform these sum in the large network limit. The first summation in Eq. (27) denotes the average loglikelihood of finding at time t a non-interacting agent given a history $\mathcal{S}_t$. We can distinguish between two eventual situations occurring at time t: *(i)* the agent has been non-interacting since a time $t-\tau$, and at time t remains non-interacting; *(ii)* the agent has been interacting with another agent since time $t-\tau$, and at time t the conversation is terminated by one of the two interacting agents. In order to characterize situation *(i)* we indicate by $P^k_{1\to1}(\tau)$ the probability that a non-interacting agent with degree k in the social network, that has not interacted since a time τ, doesn't change state. Similarly, in order to characterize situation *(ii)*, we indicate by $P^{k,k',w}_{2\to1}(\tau)$ the probability that a connected pair of agents (with degrees k and k' respectively, and weight of the link w) have interacted since time τ and terminate their conversation at time t. Given the stationary solution of the pairwise communication model, performed in the annealed approximation, the rates $P^k_{1\to1}(\tau)$ and $P^{k,k',w}_{2\to1}(\tau)$ are given by

$$
\begin{aligned}
P^k_{1\to1}(\tau) &= 1-\frac{f_1(\tau)}{N}-\frac{kf_1(\tau)}{NC}\sum_{k'}\int N_1^{k'}(\tau')f_1(\tau')d\tau' \\
&= 1-(1+ck)\frac{f_1(\tau)}{N} \\
P^{k,k',w}_{2\to1}(\tau) &= \frac{2f_2(\tau|w)}{N}
\end{aligned}
\quad (28)
$$

where the constant C is given by

$$C = \sum_{k'} \int k' N_1^{k'}(\tau') f_1(\tau') d\tau' \tag{29}$$

and $f_1(\tau)$ and $f_2(\tau|w)$ are given in Sec. 1.3. The variable $N_1^k(\tau)$ indicates the number of agents of connectivity k noninteracting since a time τ. This number can in general fluctuate but in the large network limit it converges to its mean-field value given by Eq. (10) The second term in the right hand side of Eq. (27), denotes the average loglikelihood of finding two agents in a connected pair at time t given a history $\mathcal{S}_t$. There are two possible situations that might occur for two interacting agents at time t: *(iii)* these two agents have been non-interacting, and to time t one of them decides to form a connection with the other one; *(iv)* the two agents have been interacting with each other since a time $t-\tau$, and they remain interacting at time t. To describe the situation *(iii)*, we indicate by $P_{1\to 2}^{k,k'}(\tau,\tau')$ the probability that two non interacting agents, isolated since time $t-\tau$ and $t-\tau'$ respectively, interact at time t. In order to describe situation *(iv)*, we denote by $P_{2\to 2}^{k,k',w}(\tau)$ the probability that two interacting agents, in interaction since a time $t-\tau$, remain interacting at time t. In the framework of the stationary annealead approximation of the dynamical network these probabilities are given by

$$\begin{aligned} P_{1\to 2}^{k,k'}(\tau,\tau') &= \frac{f_1(\tau)f_1(\tau')}{NC}(k+k') \\ P_{2\to 2}^{k,k',w}(\tau) &= 1-\frac{2f_2(\tau|w)}{N}. \end{aligned} \tag{30}$$

Therefore, the entropy of dynamical social networks given by Eq. (27) can be evaluated in the thermodynamic limit, and in the annealed approximation, according to the expression

$$\begin{aligned} \mathcal{S} = &- \sum_k \int_0^\infty N_1^k(\tau) P_{1\to 1}^k(\tau) \log P_{1\to 1}^k(\tau) d\tau \\ &- \sum_{k,k',w} \int_0^\infty N_2^{k,k',w}(\tau) P_{2\to 1}^{k,k',w}(\tau) \log P_{2\to 1}^{k,k',w}(\tau) d\tau \\ &- \frac{1}{2}\sum_{k,k'} \int_0^\infty \int_0^\infty N_1^k(\tau) N_1^{k'}(\tau') P_{1\to 2}^{k,k'}(\tau,\tau') \log P_{1\to 2}^{k,k'}(\tau,\tau') d\tau d\tau' \\ &- \frac{1}{2}\sum_{k,k',w} \int_0^\infty N_2^{k,k',w}(\tau) P_{2\to 2}^{k,k',w}(\tau) \log P_{2\to 2}^{k,k',w}(\tau) d\tau, \end{aligned} \tag{31}$$

with $N_1^k(\tau)$ and $N_2^{k,k',w}(\tau)$ given in the large network limit by Eqs. (10).

2.2 Entropy of the null model

To understand the impact of the distribution of duration of the interactions and of the distribution of non-interaction periods, we have compared the entropy S of the pairwise

communication model with the entropy S_R of a null model. Here we use the exponential mean-field model described in Section 1.5 as our null model. In this model the agents are embedded in a fully connected networks and the probability of changing the agent state does not include the reinforcement dynamics. In fact we have that the transition rates are independent of time ($\beta = 0$) and given by $f_1^R(\tau) = b_1^R$ and $f_2^R(\tau) = b_2^R$. Following the same steps used in Sec. 2.1 for evaluating S in the model of pairwise communication on the networks, it can be easily proved that the entropy S_R of the dynamical null model is given by

$$\begin{aligned}
S_R = & - \int_0^\infty N_1^R(\tau)\left[1-\frac{2b_1^R}{N}\right]\log\left[1-\frac{2b_1^R}{N}\right]d\tau \\
& - \int_0^\infty N_2^R(\tau)\frac{2b_2^R}{N}\log\frac{2b_2^R}{N}d\tau \\
& - \frac{1}{2}\int_0^\infty\int_0^\infty N_1^R(\tau)N_1^R(\tau')\frac{2b_1^R}{NC^R}\log\frac{2b_1^R}{NC^R}d\tau d\tau' \\
& - \frac{1}{2}\int_0^\infty N_2^R(\tau)\left[1-\frac{2b_2^R}{N}\right]\log\left[1-\frac{2b_2^R}{N}\right]d\tau \qquad (32)
\end{aligned}$$

where the constant C^R is given by

$$C^R = \int_0^\infty N_1^R(\tau)d\tau, \qquad (33)$$

and where N_1, N_2 are given , in the large network limit by their mean-field value given by Eq.(25). In order to build an appropriate null model for the pairwise communication model parametrized by (β, b_1, b_2) ,we take the parameters of the null model b_1^R and b_2^R such that the proportion of the total number of agents in the two states (interacting or non-interacting) is the same in the pairwise model of social communication and in the null model. In order to ensure this condition we need to satisfy the following relation

$$\frac{\sum_k \int_0^\infty N_1^k(\tau)d\tau}{\sum_{k,k',w}\int_0^\infty N_2^{k,k',w}(\tau)d\tau} = \frac{\int_0^\infty N_1^R(\tau)d\tau}{\int_0^\infty N_2^R(\tau)d\tau}. \qquad (34)$$

In particular we have chosen $b_1^R = b_1$ and we have used Eq. (34) to determine b_2^R.

3 Measurement of the entropy of a typical week-day of cell-phone communication from the data

In this section we discuss the method of measuring the dynamical entropy from empirical cellphone data as a function of time t in a typical weekday. This analysis gave rise to the results presented in figure 2 in the main body of the paper. We have analyzed the call sequence of subscribers of a major European mobile service provider. We considered calls between users who at least once called each other during the examined 6 months period in order to examine calls only reflecting trusted social interactions. The resulted event list consists of 633.986.311 calls between 6.243.322 users. For the entropy calculation we selected 562.337 users who executed at least one call per a day during a working week period. Since the network is very large we have assumed that the dynamical entropy can be evaluate in the mean-field approximation. We measured the following quantities directly from the sample:

- $N_1(\tau, t)$ the number of agents in the sample that at time t are not in a conversation since time $t - \tau$;
- $N^{calls}(\tau, t)$ the number of agents in the sample that are not in a conversation since time $t - \tau$ and make a call at time t;
- $N^{called}(\tau, t)$ the number of agents in the sample that are not in a conversation since time $t - \tau$ and are called at time t;
- $M^{in}(\tau, t)$ the number of agents that at time t are in a conversation of duration τ with another agent in the sample;
- $M^{out}(\tau, t)$ the number of agents that at time t are in a conversation of duration τ with another agent outside the sample;
- $M^{end}(\tau, t)$ the number of calls of duration τ that end at time t.

Using the above quantities, we estimated the probability $p^{calls}(\tau, t)$ that an agent makes a call at time t after a non-interaction period of duration τ, the probability $p^{called}(\tau, t)$ that an agent is called at time t after a non-interaction period of duration τ and the probability $\pi(\tau, t)$ that a call of duration τ ends at time t,according to the following relations

$$\begin{aligned} p^{calls}(\tau, t) &= \frac{N^{calls}(\tau, t)}{N_1(\tau, t)} \\ p^{called}(\tau, t) &= \frac{N^{called}(\tau, t)}{N_1(\tau, t)} \\ \pi(\tau, t) &= \frac{M^{end}(\tau, t)}{M^{in}(\tau, t)/2 + M^{out}(\tau, t)}. \end{aligned} \tag{35}$$

Since the sample of 562.337 users we are considering is a subnetwork of the whole dataset constituted by 6.243.322 users, in our measurement, an agent can be in one of three possible states

- *state 1:* the agent is non-interacting;
- *state 2:* the agent is in a conversation with another agent of the sample;
- *state 3:* the agent is in a conversation with an agent outside the sample.

Therefore , to evaluate the entropy of the data, we can modify Eq.(27) into

$$\begin{aligned}
\mathcal{S}(t) = & - \sum_i P(g_i(t)=1|\mathcal{S}_t)\log P(g_i(t)=1|\mathcal{S}_t) \\
& - \sum_{ij} a_{ij} P(g_{ij}(t)=1|\mathcal{S}_t)\log P(g_{ij}(t)=1|\mathcal{S}_t) \\
& - \sum_i P(g'_i(t)=1|\mathcal{S}_t)\log P(g'_i(t)=1|\mathcal{S}_t) \qquad (36)
\end{aligned}$$

where a_{ij} is the adjacency matrix of the quenched social network, $g_i(t) = 1$ indicates that the agent i is in state 1, $g_{ij}(t) = 1$ indicates that the agent is in state 2 interacting with agent j and $g'_i(t) = 1$ indicates the agent i is in state 3. Finally $\mathcal{S}_t = \{g_i(t'), g_{ij}(t')\ g'_i(t) \quad \forall t' < t\}$ indicates the dynamical evolution of the social network. To explicitly evaluate Eq. (36) in the large network limit where we assume that the dependence on the particular history are vanishing, we sum over the loglikelihood of all transitions between different states using the same strategy in Sec.2, which is

$$\begin{aligned}
\mathcal{S}(t) = & - \sum_\tau N_1(\tau,t)P_{1\to1}(\tau,t)\log P_{1\to1}(\tau,t) \\
& - \sum_\tau M^{in}(\tau,t)P_{2\to1}(\tau,t)\log P_{2\to1}(\tau,t) \\
& - \sum_\tau M^{out}(\tau,t)P_{3\to1}(\tau,t)\log P_{3\to1}(\tau,t) \\
& - \frac{1}{2}\sum_{\tau,\tau'} N_1(\tau,t)N_1(\tau',t)P_{1\to2}(\tau,\tau',t)\log P_{1\to2}(\tau,\tau',t) \\
& - \frac{1}{2}\sum_\tau M^{in}(\tau,t)P_{2\to2}(\tau,t)\log P_{2\to2}(\tau,t) \\
& - \sum_\tau N_1(\tau,t)P_{1\to3}(\tau,t)\log P_{1\to3}(\tau,t) \\
& - \sum_\tau M^{out}(\tau,t)P_{3\to3}(\tau,t)\log P_{3\to3}(\tau,t). \qquad (37)
\end{aligned}$$

where the probabilities of transitions between different states are given by

$$\begin{aligned}
P_{1\to1}(\tau,t) &= 1-p^{calls}(\tau,t)-p^{called}(\tau,t)\\
P_{2\to1}(\tau,t) &= P_{3\to1}(\tau,t)=\pi(\tau,t)\\
P_{1\to2}(\tau,\tau',t) &= \frac{(1-\gamma)}{C}\left[p^{calls}(\tau,t)p^{called}(\tau',t)+p^{calls}(\tau',t)p^{called}(\tau,t)\right]\\
P_{2\to2}(\tau,t) &= P_{3\to3}(\tau,t)=1-\pi(\tau,t)\\
P_{1\to3}(\tau,t) &= \gamma\left[p^{calls}(\tau,t)+p^{called}(\tau,t)\right] \qquad (38)
\end{aligned}$$

and where C is given by

$$C=\sum_{\tau} N_1(\tau,t)p^{called}(\tau,t). \qquad (39)$$

Finally in 38 we have introduced a parameter $\gamma \in [0,1]$ to denote the portion of the calls occurring between an agent in the sample and an agent out of the sample. For simplicity, we assume that γ is a constant. Substituting Eq.(38) into Eq.(37), we have performed the summation over τ to obtain the value of entropy as a function of t presented in Figure 2 of the main body of the paper where we have taken $\gamma = 0.8$, consistently with the data.

References

[1] Zhao K, Stehlé J, Bianconi G, Barrat A (2011) Social network dynamics of face-to-face interactions. *Phys Rev E* 83:056109.
